# Quantitative evaluation of protocorm growth and fungal colonization in *Bletilla striata* (Orchidaceae) reveals less-productive symbiosis with a non-native symbiotic fungus

**DOI:** 10.1186/s12870-017-1002-x

**Published:** 2017-02-21

**Authors:** Tatsuki Yamamoto, Chihiro Miura, Masako Fuji, Shotaro Nagata, Yuria Otani, Takahiro Yagame, Masahide Yamato, Hironori Kaminaka

**Affiliations:** 1 Graduate School of Agriculture, Tottori University, Tottori, Japan; 2 Faculty of Agriculture, Tottori University, Tottori, Japan; 3 Tsukuba Botanical Garden, National Museum of Nature and Science, Tsukuba, Japan; 4 Faculty of Education, Chiba University, Chiba, Japan

**Keywords:** *Bletilla striata*, Germination, Mycorrhizal symbiosis, Orchid, Quantitative evaluation

## Abstract

**Background:**

In nature, orchid plants depend completely on symbiotic fungi for their nutrition at the germination and the subsequent seedling (protocorm) stages. However, only limited quantitative methods for evaluating the orchid–fungus interactions at the protocorm stage are currently available, which greatly constrains our understanding of the symbiosis. Here, we aimed to improve and integrate quantitative evaluations of the growth and fungal colonization in the protocorms of a terrestrial orchid, *Blettila striata*, growing on a plate medium.

**Results:**

We achieved both symbiotic and asymbiotic germinations for the terrestrial orchid *B. striata*. The protocorms produced by the two germination methods grew almost synchronously for the first three weeks. At week four, however, the length was significantly lower in the symbiotic protocorms. Interestingly, the dry weight of symbiotic protocorms did not significantly change during the growth period, which implies that there was only limited transfer of carbon compounds from the fungus to the protocorms in this relationship. Next, to evaluate the orchid–fungus interactions, we developed an ink-staining method to observe the hyphal coils in protocorms without preparing thin sections. Crushing the protocorm under the coverglass enables us to observe all hyphal coils in the protocorms with high resolution. For this observation, we established a criterion to categorize the stages of hyphal coils, depending on development and degradation. By counting the symbiotic cells within each stage, it was possible to quantitatively evaluate the orchid-fungus symbiosis.

**Conclusions:**

We describe a method for quantitative evaluation of orchid-fungus symbiosis by integrating the measurements of plant growth and fungal colonization. The current study revealed that although fungal colonization was observed in the symbiotic protocorms, the weight of the protocorm did not significantly increase, which is probably due to the incompatibility of the fungus in this symbiosis. These results suggest that fungal colonization and nutrition transfer can be differentially regulated in the symbiosis. The evaluation methods developed in this study can be used to study various quantitative aspects of the orchid-fungus symbiosis.

**Electronic supplementary material:**

The online version of this article (doi:10.1186/s12870-017-1002-x) contains supplementary material, which is available to authorized users.

## Background

Orchidaceae is the largest plant family, comprising an estimated 25,000 species [1], which form mycorrhizae with a wide range of fungi in the Basidiomycota and Ascomycota. Orchid plants produce numerous minute seeds with little nutrient storage [2] and depend on symbiotic fungi for nutrition including carbon compounds during the early seedling (protocorm) stage. This nutritional mode, in which plants rely on symbiotic fungi, is termed mycoheterotrophy [3]. Mycorrhizal symbiosis in the orchid protocorm is established as follows: a hypha enters a parenchyma cell, branches to form dense hyphal coils called pelotons, and is ultimately degraded [3, 4]. It is generally accepted that carbon transfer from the fungus to the protocorm mainly occurs during peloton degradation [5], suggesting that orchids parasitize their symbiotic fungi at the protocorm stage. Although this parasitism is a cornerstone in the life strategy of orchids [6], the mechanism underlying the interaction with their symbiotic fungi remains to be elucidated.

In previous studies of symbiotic orchid protocorms, germination rate, developmental stage, size, and volume of protocorms have been used as evaluation criteria [7–16]. In addition, internal hyphal coils have also been observed to evaluate the symbiotic potential of orchid-mycorrhizal fungus [4, 17–21]. In particular, Hadley and Williamson evaluated the number of pelotons and the volume of protocorms in the symbiosis of *Dactylorhiza purpurella*, revealing a positive correlation between the two parameters [22]. Although various stages of hyphal coils, from development to degradation, are observed in symbiotic orchid protocorms [3, 4], few studies have focused on the effect of the stages of hyphal coils on protocorm growth.

A terrestrial orchid, *Bletilla striata*, known as a common garden plant in Japan [23], grows rapidly and produces numerous seeds. These features make *B. striata* a potentially useful model species for orchid-mycorrhizal research. Masuhara and Katsuya [23] reported effects of mycorrhizal fungi on *B. striata* seed germination and protocorm growth, and evaluated the symbiosis using seed germination rate and length of the protocorm. These authors also assessed the symbiosis based on observations of hyphal coils in the protocorm. However, we need more detailed quantitative evidence for the effects of symbiosis, especially regarding what occurs inside of the symbiotic cells to understand the mechanism underlying orchid–fungus interactions.

In this study, we developed a method for quantifying protocorm growth and fungal colonization during *B. striata*–fungus symbiosis. First, we achieved apparent synchronous germination with or without symbiotic fungi in *B. striata* to compare fungus-dependent and -independent growth. For protocorm growth analysis, we measured dry weight as well as length. Moreover, we developed a staining procedure to assess the number of symbiotic cells and quantitatively evaluate the development and degradation of hyphal coils. Our method enabled us to observe the details of all hyphal coils. By integrating the measurements, we could evaluate the effects of symbiosis in detail. Finally, we discuss prospects for future studies aimed at understanding the mechanisms underlying this symbiosis.

## Methods

### Plant material and isolation of symbiotic fungi

Seeds of *B. striata* ‘Murasakishikibu’ collected five months after self-pollination of plants purchased from a garden store were used in this study. The *B. striata* strain ‘Murasakishikibu’ was originally selected as a specific flower-color variant from a habitat in Miyazaki Prefecture, Japan, and has been maintained for more than 20 years by gardeners.

The symbiotic fungus was isolated from roots of *Pecteilis radiata* (Thunb.) Raf. collected with owner’s permission on Aug. 3, 2003 at private land in Himeji, Hyogo Prefecture, Japan. The habitat of this orchid was a rough wetland, where the place had been maintained as a paddy field until 10 years ago. Mycobiont of this orchid was isolated according to the method of Warcup and Talbot [24] with slight modifications as follows. The surface of the root was washed with tap water and sterilized by immersion in 70% ethanol for 30 s and in sodium hypochlorite solution containing 1% available chlorine for 30 s. The surface-sterilized root was then cut into small pieces approximately 10 mm long. The pieces were placed into a Petri dish (9 cm diameter) with 1 ml sterilized distilled water and crushed with a sterilized glass rod to disperse the intracellular hyphal coils (pelotons). Autoclaved modified Czapek Dox agar (0.5 g sucrose, 0.33 g NaNO_3_, 0.2 g KH _2_ PO_4_, 0.1 g MgSO_4_ · 7H _2_ O, 0.1 g KCl, 0.1 g yeast extract, 15 g agar, 1 l distilled water) was cooled to 45 °C and poured into the Petri dishes (~20 ml per dish). The dishes were mixed well before solidification to disperse the pelotons throughout the medium. The plates were incubated at 25.0 ± 0.5 °C in the dark for 3 d. Fungal colonies growing from the pelotons were isolated using a sterilized scalpel and cultivated on potato dextrose agar (PDA, Difco, Franklin, New Jersey, USA) medium. One of the fungal isolates, HR1-1, was used for symbiotic germination in this study.

### Phylogenetic analysis

DNA was extracted from the isolated fungus using PrepMan Ultra Reagent (Applied Biosystems, Foster City, California, USA) according to the manufacturer’s instructions. The ITS of rDNA was amplified from the extracted DNA by PCR with the primers ITS1-OF/ITS4-OF [25] using TaKaRa Ex Taq Hot Start Version (Takara Bio, Otsu, Japan). The PCR mixture contained 5 μl template DNA, 0.75 units Taq polymerase, 0.25 μmol/l each primer, 200 μmol/l each dNTP, and 3 μl of the supplied PCR buffer in a total volume of 30 μl. The amplification of the ITS region was performed on a PC-818S Program Temp Control System (Astec, Fukuoka, Japan) as follows: initial denaturation at 94 °C for 2 min followed by 35 cycles of 94 °C for 20 s, 55 °C for 30 s, and 72 °C for 1 min and a final elongation step at 72 °C for 5 min. PCR products were cloned using the pGEM-T Easy Vector System I (Promega, Tokyo, Japan), and plasmid DNAs were extracted from the cloned products using MagExtractor Plasmid (TOYOBO). The plasmid inserts were sequenced using the dye terminator method with sequencing primers T7 and SP6. All sequences were subjected to BLAST searches [26], and the related sequences were downloaded from the DDBJ/EMBL/GenBank nucleotide sequence database. Sequence alignment was performed using the CLUSTAL W program [27]. For phylogenetic analyses, neighbor-joining analysis [28] was performed with MEGA version 5 [29] with bootstrap analysis of 1000 replications [30]. Evolutionary distances were estimated using γ–distributed rates. The phylogenetic tree was drawn with TreeView software [31].

### Symbiotic and asymbiotic germination

The seeds were surface sterilized in sodium hypochlorite with 1% available chlorine concentration containing 0.05% Tween 80 for 2 min and rinsed with sterilized water. Approximately 50 sterilized seeds were placed into plates containing either 20 ml original (1×), double (2×), or quadruple (4×) strength of oatmeal agar medium (2.5 g, 5.0 g, or 10.0 g, respectively, of oatmeal agar [Difco, Franklin, New Jersey, USA], 6.5 g agar, 1 l distilled water, pH 5.5) with symbiotic fungus which is precultured on 1× oatmeal agar medium for a week at 25 °C for symbiotic germination or 20 ml Hyponex agar medium (3.0 g Hyponex [6.5–6-19] [Hyponex Japan, Osaka, Japan], 2.0 g peptone, 30 g sucrose, 10 g agar, 1 l distilled water, pH 5.5) for asymbiotic germination. The germination experiments were conducted at 25 °C in the dark, and several randomly chosen protocorms were collected every seven days for four weeks. At each protocorm collection, images of the protocorms were taken under an SZX16 stereomicroscope (Olympus, Tokyo, Japan).

### Protocorm growth measurements

The length and width of the collected protocorms were measured using the following procedure with Image J software version 1.47 (http://imagej.nih.gov/ij) as shown in Additional file 1: (1) a straight line (broken line) was drawn from the basal end to the apical end of the protocorm to measure the length (L), (2) a straight line (solid line) was drawn through both ends of the swollen embryo, and (3) a straight line (dotted line) was drawn perpendicular to the solid line at the most swollen site to measure the width (W). Three protocorms were measured at each sampling time point, and each experiment was repeated five times.

After rinsing the protocorms with distilled water, 10 protocorms were placed in a single **∅**5 × 19 mm tin capsule (Ludi Swiss AG, Switzerland) and dried for 1 week at 60 °C. The dry weights were then measured using a microbalance (Mettler Toledo, Columbus, OH). Three independent germination experiments were performed for the dry weight measurements.

### Quantitative evaluation of seed germination

The number of germinated seeds on oatmeal agar medium or Hyponex agar medium was counted under an SZX16 stereomicroscope (Olympus, Tokyo, Japan). At least 50 seeds were observed for each germination method, in which germination was defined as the emergence of a rhizoid or shoot. Three independent germination experiments were performed for the measurements of the germination rates.

### Ink staining of hyphal coils in protocorm

The germinated symbiotic protocorms were stored in FAA solution at 4 °C for subsequent quantitative evaluation of fungal colonization. The FAA-fixed protocorms were rinsed with distilled water through a 40-μm cell strainer (Corning, NY, USA) and autoclaved at 121 °C for 20 min in 10% (w/v) KOH solution. The autoclaved protocorms were neutralized in 2% (v/v) HCl for 5 min, transferred to 10% (v/v) ink dye solution (10% Pelikan 4001 Brilliant Black and 3% acetic acid), heated at 95 °C for 30 min, and soaked in 100% lactic acid (Nakarai tesque Inc., Kyoto, Japan) at 4 °C before microscopic observation.

### Quantitative evaluation of fungal colonization

The stained protocorms were processed as shown in Additional file 2. The testa of the stained protocorm was removed using a dissecting needle under an SZX16 stereomicroscope (Olympus, Tokyo, Japan), and the protocorm was transferred to a glass slide. The protocorm was covered with a cover glass and crushed using the end of the grip of a dissecting needle. The number of symbiotic cells was counted under a BX53 light microscope (Olympus, Tokyo, Japan) in at least 10 different protocorms at each sampling time. Three independent germination experiments were performed for this quantitative evaluation.

### Staining hyphae on oatmeal agar medium

Hyphae of the symbiotic fungus on oatmeal agar medium were stained for two hours in a solution of 0.05% Trypan blue in lactic acid at room temperature. The hyphae were washed three times with distilled water. Stained hyphae were observed under an SZX16 stereomicroscope (Olympus, Tokyo, Japan). Five replicate plates were prepared for each concentration of oatmeal medium (1×, 2×, and 4×).

## Results and discussion

### Symbiotic and asymbiotic germination of *B. striata*

Masuhara et al. [32] isolated mycorrhizal fungi from roots of *B. striata*, and most of the fungi were identified as *Rhizoctonia repens*. This anamorphic fungal species is one of the representative mycorrhizal fungi in terrestrial orchids, and some of fungi isolated from *Goodyera schlechtendaliana*, *Spiranthes sinensis*, *Dendrobium nobile*, *Ponerorchis kurokamiana*, and *P. graminifolia* induce symbiotic germinations of *B. striata* under in vitro condition [23]. Though our previous attempts to isolate mycorrhizal fungi from *B. striata* roots were unsuccessful, we achieved symbiotic germination of *B. striata* with one of the fungal isolates, HR1-1, obtained from roots of *Pecteilis radiata*. This fungus was identified as *Tulasnella* sp. (Tulasnellaceae) as described below, and it is well known that the teleomorph of *Rhizoctonia repens* is *Tulasnella* sp. [33]. Accordingly, we chose to use this fungus as the symbiotic fungus in this study.

**Fig. 1:**
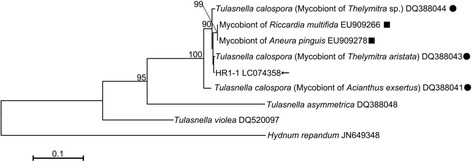
Phylogenic relationship of *Pecteilis radiata* isolate-related fungi. Neighbor-joining phylogenetic tree showing the relationship between the fungal isolate HR1-1 from *P. radiata* (*arrow*) utilized in this study and related fungi in Tulasnellaceae based on the sequences of the ITS region of nuclear rDNA. *Hydnum repandum* (JN649348) was used as the outgroup species. All bootstrap values are above 80% (1000 replicates). Accession numbers from the GenBank nucleotide database are given for all sequences. *Circles* and *squares* indicate orchid mycobionts and liverwort endophytes, respectively

The strain HR1-1 showed the highest identity with *Tulasnella calospora* DQ388043, a mycobiont of the orchid *Acianthus exsertus* (Fig. 1). In the symbiotic germination experiment, rhizoid formation, the first differentiation step in the seed germination of *B. striata*, was found in 44.9% of the seeds sown in one week, a rate higher and faster than those of terrestrial orchid species in previous symbiotic germination tests [12, 34, 35] (see Additional file 3). This result indicates that our method for symbiotic germination is appropriate for *B. striata*. In addition, we performed asymbiotic germination using Hyponex agar medium, which is a nutrient-rich medium, to evaluate the growth potential of the germinated protocorms. Rhizoids were observed in 58.1% of the seeds on asymbiotic germination medium within one week. Consequently, we could assess the effects of symbiosis by directly comparing the initial growth rates of symbiotic versus asymbiotic protocorms.

### Quantitative evaluation of the initial growth of *B. striata* protocorms

**Fig. 2:**
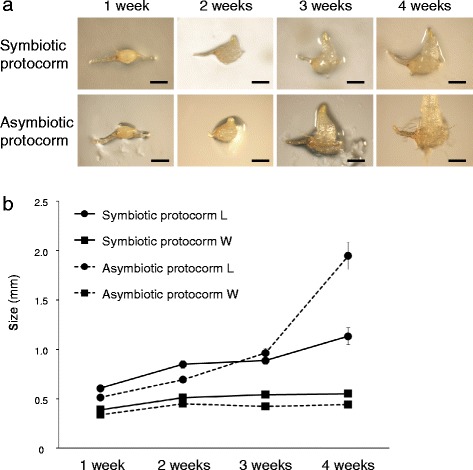
*Bletilla striata* protocorms in symbiotic and asymbiotic culture. **a** *B. striata* protocorms cultured on oatmeal ager medium with symbiotic fungus HR1-1 (Symbiotic protocorm) and on Hyponex agar medium without the symbiotic fungus (Asymbiotic protocorm). Images were collected every week for 4 weeks. Scale bars, 500 μm. **b** The length and width of symbiotic and asymbiotic protocorms. Filled *circles* and *squares* indicate length (L) and width (W), respectively. *Solid* and *broken lines* represent symbiotic and asymbiotic protocorms, respectively. *Error bars* represent the standard errors of the mean of five replicates

The symbiotic and asymbiotic protocorms grew synchronously for the first three weeks, and then asymbiotic protocorms appeared to actively grow compared to symbiotic protocorms (Fig. 2a). To quantify the size of protocorms, we first tried to measure the length and width according to the method for *Dactylorhiza purpurella* by Hadley and Williamson [22]. However, this method was not appropriate for *B. striata* because the protocorm morphology was different from that of *D. purpurella*. The width of *B. striata* protocorms did not increase much during growth (Fig. 2b). We therefore sought to establish a more appropriate method for measuring the size of *B. striata* protocorms. We defined the length of the protocorm as the linear distance between its basal and apical ends (see Additional file 1), because the swollen embryo extended perpendicularly from the major axis of ellipse. For the first three weeks, the protocorm length was almost the same under symbiotic and asymbiotic germination (Fig. 2b). After four weeks, however, the length of asymbiotic protocorms was significantly larger than that of the symbiotic protocorms. Accordingly, we used protocorm length as an index of evaluation for the initial growth of *B. striata*.

**Fig. 3:**
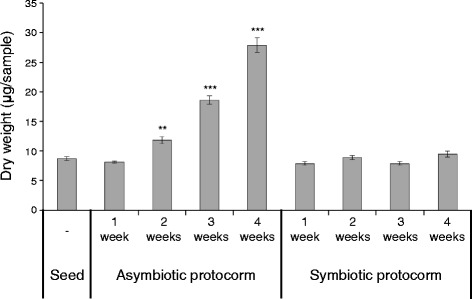
Dry weight of symbiotic and asymbiotic protocorms. Dry weight of symbiotic and asymbiotic protocorms at one to four weeks after seeding. *Error bar* represents the standard errors of the mean of three replicates. Welch *t*-test was used to identify statistically significant differences compared with the dry weights of seeds (**: *p* < 0.01, ***: *p* < 0.001)

As another method for growth evaluation, we determined the dry weight of the protocorms. The dry weight of the asymbiotic protocorms sharply increased over time, from 8.1 μg at one week to 27.9 μg at four weeks (Fig. 3). However, unexpectedly, the dry weight of symbiotic protocorms did not significantly change during (at least) the first four weeks (Fig. 3). The swollen symbiotic protocorms with less increase in dry weight may suggest that water uptake is enhanced in the symbiosis. This result is inconsistent with the previous finding that the dry weight of symbiotic germinated protocorms of *D. purpurella* harboring the mycorrhizal fungi *Rhizoctonia* spp. was approximately twice as high as that of the non-inoculated control within four weeks of growth [17]. To explore this further, we tested whether the lack of weight increase of the symbiotic protocorms may be due to the depletion of the organic carbon source in the oatmeal agar medium used in the current study. We investigated symbiotic germination using the same procedure with 2× and 4× oatmeal agar. The protocorm length was 1.14–1.55 fold greater on the media with the higher concentrations of oatmeal (see Additional file 4a), on which the hyphal density of the symbiotic fungi was also greater (see Additional file 5). Unexpectedly, the dry weights of *B. striata* protocorms were not significantly different among the 1×, 2×, and 4× oatmeal media (see Additional file 4b). Meanwhile, a previous study showed that protocorm weight is positively correlated with protocorm length [34]. Our results indicate that the carbon source in the oatmeal agar medium had no significant effect on protocorm weight, while there was a positive correlation between the amount of the carbon source and protocorm length, suggesting that there is limited transfer of the carbon compounds from the strain HR1-1 to *B. striata* protocorms in the symbiotic relationship on the medium. These results indicate that dry weight is also an important index of evaluation for the growth of the symbiotic protocorms.

### Quantitative evaluation of symbiosis in *B. striata* protocorms

**Fig. 4:**
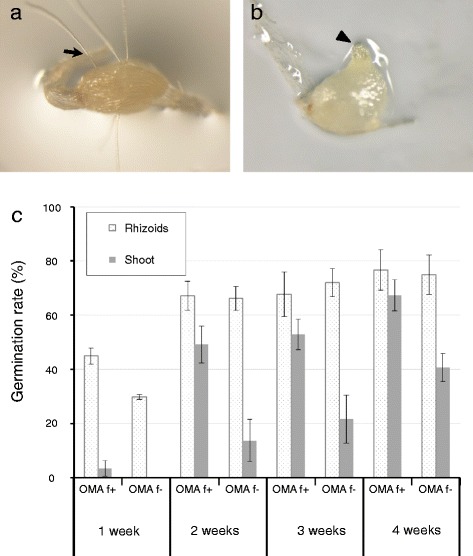
Germination criteria and rates in symbiotic germinated protocorms. **a** *B. striata* protocorm with swollen embryo and rhizoids, as denoted by the arrow. **b** Protocorm with a shoot apex (protomeristem), as denoted by the arrowhead. **c** Germination rates of *B. striata* protocorms on oatmeal agar medium with symbiotic fungus HR1-1 (OMA f+) or without fungi (OMA f-). The *dotted* and *gray bars* indicate the germination rates using different criteria, i.e., the appearance of a rhizoid or shoot apex, respectively. *Error bars* represent standard errors of the mean of three replicates

Many studies have described the symbiotic germination of terrestrial orchid seeds with their associated fungi in vitro using germination rate as an index of symbiotic efficiency [7–9, 12–16]. Like these previous studies, we initially tried to use the production of rhizoids as the criterion for measuring germination rates in symbiotic germination of *B. striata* seeds (Fig. 4a). However, the conventional criteria for germination were not suitable for *B. striata*, because the seeds formed rhizoids on oatmeal agar medium in both the presence and absence of fungal hyphae. To overcome this problem, we introduced another criterion, the appearance of the shoot apex (protomeristem), as an indicator of *B. striata* seed germination (Fig. 4b). Using this criterion, the germination rate with symbiotic fungi was significantly higher than that without fungi at all time points on oatmeal agar medium (Fig. 4c). This result suggests that one- to three-week-old protocorms, which showed the most marked differences in germination in the presence versus absence of symbiotic fungi, are well suited for studying the physiological aspects of symbiotic associations between symbiotic fungi and *B. striata*. However, a maximum of 40.7% of seeds also had shoot apices on the oatmeal agar medium even without symbiotic fungi, indicating that only one parameter, e.g., seed germination rate, seems to be not sufficient to evaluate this symbiosis.

**Fig. 5:**
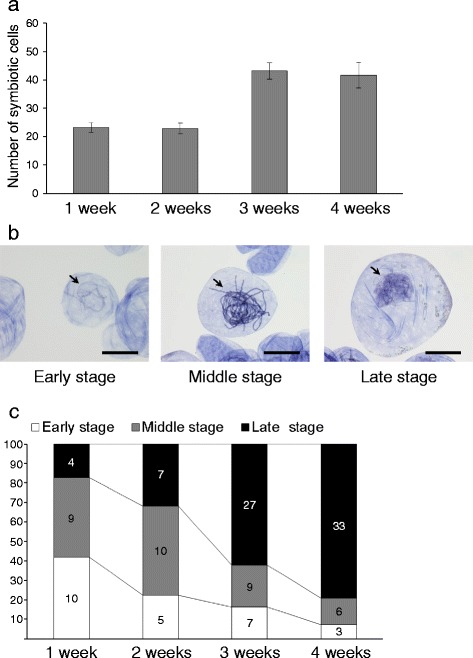
Quantification and characterization of symbiotic cells per protocorm. **a** The number of symbiotic cells per symbiotic protocorm at one to four weeks after seeding. *Error bar* represents the standard error of the mean for ten protocorms. **b** Symbiotic cells with hyphal coils at the Early, Middle, and Late stages. Scale bars, 50 μm. **c** Ratio of the number of symbiotic cells at each stage in a symbiotic protocorm. Each value represents the average number of symbiotic cells in ten protocorms. The experiment was repeated three times with similar results

For characterization of symbiosis in orchid protocorms, many studies have employed observation of internal hyphae using a resin-embedded method [17, 18, 20, 23] or a trypan blue-staining method [19, 21, 35]. The use of an improved ink-staining technique and crushing the protocorms allowed us to clearly and easily visualize the fungal hyphae in symbiotic protocorms and to count the total number of symbiotic cells containing pelotons by light microscopy (see Additional file 2). The average total number of symbiotic cells per protocorm is shown in Fig. 5a. Symbiotic cells with hyphal coils were already present by week one. There were approximately twice as many symbiotic cells on week three compared to week two. Thus, our observation method can be used to quantify the symbiotic cells in *B. striata* protocorms with high resolution and accuracy.

In addition, to analyze the correlation between the developmental stages of hyphal coils and the germination or initial growth of *B. striata*, we classified the hyphal coils into three stages according to fungal morphological characteristics (Fig. 5b): Early stage, characterized by fungal invasion and initiation of hyphal coiling; Middle stage, characterized by well-developed hyphal coils with a clear fungal cell wall; Late stage, characterized by disruption of fungal septa via degradation of the hyphal coils. We determined the ratio of hyphal coils at each stage per protocorm. The ratios of hyphal coils at the Early or Middle stage were 82.7 and 68.1% on weeks one and two, respectively (Fig. 5c). By contrast, the ratio of hyphal coils at the Late stage increased at week three (62.2%) and four (79.2%; Fig. 5c). These results indicate that the germination stimulus is induced during the Early or Middle stage of symbiosis before the increase in peloton degradation. These findings are consistent with those regarding the symbiosis between *D. purpurella* and *Ceratobasidium*s, in which the growth stimulus may be independent of an external supply of carbohydrate [22].

To test the applicability of our observation methods to other species, *P. radiata* seeds were inoculated with the strain HR1-1 using the same symbiotic germination method. In this experiment, rhizoids were observed in 19.1% of seeds and the three stages of symbiotic cells were also easily observed, as in a *B. striata* protocorms, at two weeks (see Additional file 6). Thus, our staining and quantification methods are suitable for other species beyond *B. striata*.

As described above, the number of symbiotic cells in protocorms grown on 2× and 4× oatmeal consistently increased during the four week growth period and tended to be higher in a concentration-dependent manner (see Additional file 7). However, the dry weights of the symbiotic protocorms were not correlated with the number of symbiotic cells and/or the increase of the cells with degraded hyphal coils (see Additional file 7). Recently, Kuga et al. [4] reported that carbon is mostly transferred from degraded hyphal coils to orchid protocorms; however, the development and degradation of the hyphal coils seemed to not affect protocorm growth during the symbiosis under our experimental conditions. This lack of effect on protocorm growth suggests that nutrient transfer does not take place efficiently in this symbiosis.

## Conclusion

In this study, we established a method for quantitative evaluation of orchid symbiosis by measuring length and weight of protocorms and the number of symbiotic cells in different stages. This method can be used to study physiological aspects of orchid–fungus interactions such as compatibility, one of the major concerns in orchid symbiosis. In fact, we were able to demonstrate that the orchid *B. striata* formed a symbiosis with *Tulasnella* sp. strain HR1-1, but that the weight of symbiotic protocorms did not significantly change during the study period, even when more symbiotic cells were found in the protocorms. These results indicated that there was little net transfer of carbon compounds from HR1-1 to *B. striata* protocorms, which is probably due to incompatibility between the orchid and the fungus in this symbiosis. We observed no abnormalities in the characteristics of the fungal colonization, in terms of development and degradation of hyphal coils observed with our ink-staining technique. Taken together, our results imply that fungal colonization and nutrition transfer from fungus to orchid may be differentially regulated during orchid symbiosis. Further study is needed to quantify *B. striata* growth and symbiosis with its native symbiotic partner and to assess carbon transfer during that symbiosis.

With very little modification, the method in this study will be widely applicable to other orchid species. In addition to in vitro germination studies, the current method could also be applicable to use for field studies like that of Stöckel et al. [36], who examined in the field carbon and nitrogen stable isotope compositions in seedlings and adults of orchids to evaluate the growth response. Such studies will help increase our understanding of the mechanisms underlying orchid–fungus symbiosis. The specificity between orchids and mycorrhizal fungi has been the subject of controversy for a long time [37]. Green terrestrial orchids likely exhibit low fungal specificity at the germination stage [38, 39]. By contrast, Warcup and other researchers have reported that at least genus-level specificity of symbiotic fungi might exist [7, 40, 41]. Our quantitative method for analysis of fungal colonization could be used to help resolve these conflicting views of various orchids with accurate data.

## Additional files

Additional file 1: Measuring procedure for length and width of symbiotic and asymbiotic protocorm. The broken line shows a protocorm length (L). The solid line is drawn through the both ends of the swollen embryo. The dotted line shows a protocorm width (W), which is perpendicular to the solid line at the most swollen site. (PDF 62 kb)
Additional file 2: Sample preparation for quantitative evaluation of symbiotic cells in a protocorm. (a) A stained protocorm with ink solution. (b) A protocorm with testa removed. (c) Application of pressure to disassemble protocorm cells into the single layer. (d) Disassembled cells of a protocorm. Scale bars, 500 μm. (PDF 530 kb)
Additional file 3: Germination rates of several orchid species. (PDF 58 kb)
Additional file 4: Length and dry weight of symbiotic protocorm under the conditions with high concentration of oatmeal. (a) The length of symbiotic protocorms under the 2×- and 4×-strength oatmeal for four weeks after seeding. (b) The dry weight of symbiotic protocorm under the 2×- and 4×-strength oatmeal conditions for four weeks after seeding. Error bars of (a) and (b) represent the standard errors of the mean in five and three biological replicates, respectively. (PDF 124 kb)
Additional file 5: Growth of fungal hyphae on oatmeal agar medium. The hyphae of the symbiotic fungi on oatmeal agar media with (a) 1×-, (b) 2×-, and (c) 4×- strength oatmeal stained by 0.05% trypan blue solution. Scale bars, 500 μm. The experiment was repeated five times with similar results. (PDF 977 kb)
Additional file 6: Quantitative evaluation of symbiotic cells in *Pecteilis radiata* protocorm. (a) Symbiotic cells with hyphal coils in *P. radiata* protocorm. Scale bars, 50 μm. (b) Ratio of the number of symbiotic cells at each stage in a symbiotic protocorm. Each value represents the average number of symbiotic cells in ten protocorms. The experiments were repeated six times with similar results. (PDF 959 kb)
Additional file 7: Quantitative evaluation of symbiotic cells in a protocorm under conditions with high concentrations of oatmeal. (a) The number of symbiotic cells per one protocorm under 2×- and 4×-strength oatmeal conditions for four weeks after seeding. (b) Ratio of the number of symbiotic cells at each stage in a symbiotic protocorm under 2×- and 4×-strength oatmeal conditions. Error bars represents the standard error of the mean in ten protocorms. The experiments were repeated three times with similar results. (PDF 140 kb)

## Abbreviations

FAA: Formalin-acetic acid-alcohol
ITS: Internal transcribed spacer
rDNA: ribosomal DNA
