# Supplementary material for: Quantitative evaluation of protocorm growth and fungal colonization in Bletilla striata (Orchidaceae) reveals less-productive symbiosis with a non-native symbiotic fungus
Source: BMC Plant Biol. 2017 Feb 21;17:50. doi: 10.1186/s12870-017-1002-x (PMC5320772; doi:10.1186/s12870-017-1002-x)
Supplement: Additional file 1: — Measuring procedure for length and width of symbiotic and asymbiotic protocorm. The broken line shows a protocorm length (L). The solid line is drawn through the both ends of the swollen embryo. The dotted line shows a protocorm width (W), which is perpendicular to the solid line at the most swollen site. (PDF 62 kb) [file 12870_2017_1002_MOESM1_ESM.pdf]

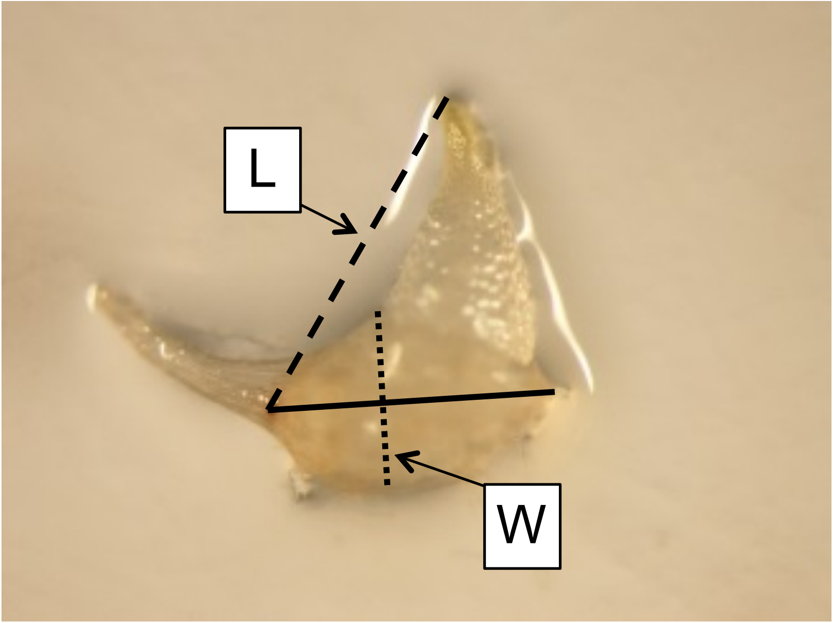

Additional file 1. Measuring procedure for length and width of symbiotic and asymbiotic protocorm.
The broken line shows a protocorm length (L). The solid line is drawn through the both ends of the swollen embryo. The dotted line shows a protocorm width (W), which is perpendicular to the solid line at the most swollen site.
