# Supplementary material for: Quantitative evaluation of protocorm growth and fungal colonization in Bletilla striata (Orchidaceae) reveals less-productive symbiosis with a non-native symbiotic fungus
Source: BMC Plant Biol. 2017 Feb 21;17:50. doi: 10.1186/s12870-017-1002-x (PMC5320772; doi:10.1186/s12870-017-1002-x)
Supplement: Additional file 2: — Sample preparation for quantitative evaluation of symbiotic cells in a protocorm. (a) A stained protocorm with ink solution. (b) A protocorm with testa removed. (c) Application of pressure to disassemble protocorm cells into the single layer. (d) Disassembled cells of a protocorm. Scale bars, 500 μm. (PDF 530 kb) [file 12870_2017_1002_MOESM2_ESM.pdf]

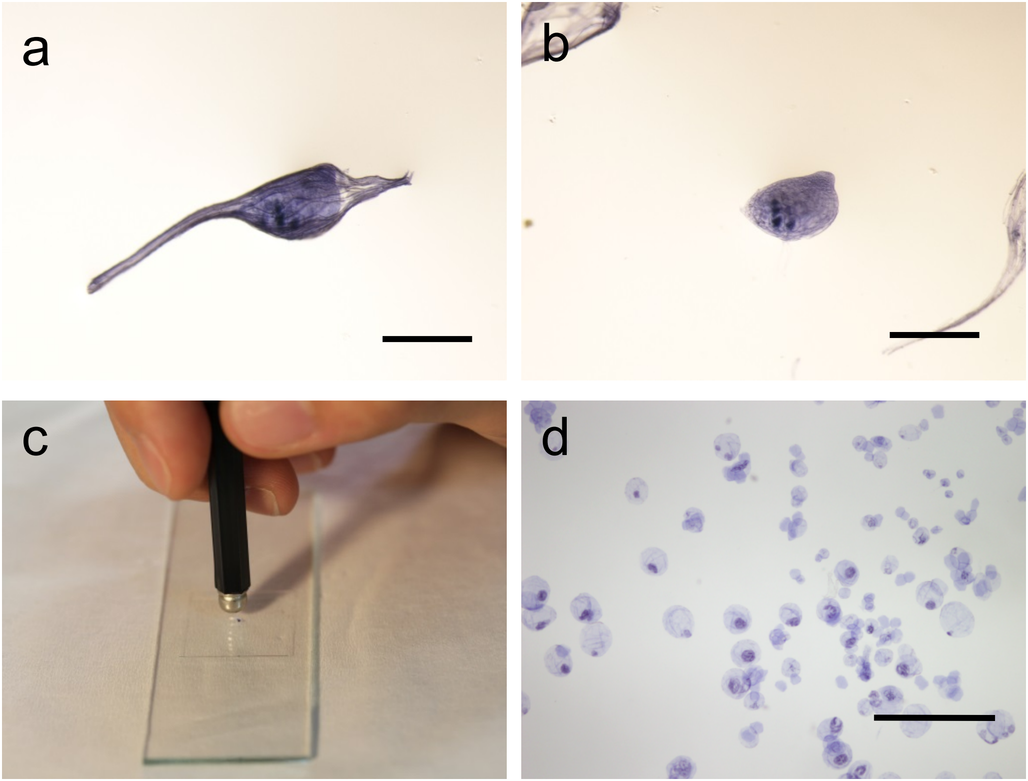

Additional file 2. Sample preparation for quantitative evaluation of symbiotic cells in a protocorm.

(a) A stained protocorm with ink solution. (b) A protocorm with testa removed. (c) Application of pressure to disassemble protocorm cells into the single layer. (d) Disassembled cells of a protocorm. Scale bars, 500 μm.
