# Supplementary material for: Quantitative evaluation of protocorm growth and fungal colonization in Bletilla striata (Orchidaceae) reveals less-productive symbiosis with a non-native symbiotic fungus
Source: BMC Plant Biol. 2017 Feb 21;17:50. doi: 10.1186/s12870-017-1002-x (PMC5320772; doi:10.1186/s12870-017-1002-x)
Supplement: Additional file 3: — Germination rates of several orchid species. (PDF 58 kb) [file 12870_2017_1002_MOESM3_ESM.pdf]

Additional file 3. Germination rates of several orchid species.

Host	Germination rate (%)	Period (days)
Bletilla striata[a]	44.9	7
Habenaria quinquiseta[b]	18.1	68
Caladenia latifolia[c]	20	14
Gavilea australis[d]	45	35
Coelogyne nervosa[e]	6	15

[a]In this study; [b]Stewart et al. [12]; [c]Bustam et al. [16]; [d]Fracchia et al. [39]; [e]Sathiyadash et al. [21]
