# Supplementary material for: Quantitative evaluation of protocorm growth and fungal colonization in Bletilla striata (Orchidaceae) reveals less-productive symbiosis with a non-native symbiotic fungus
Source: BMC Plant Biol. 2017 Feb 21;17:50. doi: 10.1186/s12870-017-1002-x (PMC5320772; doi:10.1186/s12870-017-1002-x)
Supplement: Additional file 4: — Length and dry weight of symbiotic protocorm under the conditions with high concentration of oatmeal. (a) The length of symbiotic protocorms under the 2×- and 4×-strength oatmeal for four weeks after seeding. (b) The dry weight of symbiotic protocorm under the 2×- and 4×-strength oatmeal conditions for four weeks after seeding. Error bars of (a) and (b) represent the standard errors of the mean in five and three biological replicates, respectively. (PDF 124 kb) [file 12870_2017_1002_MOESM4_ESM.pdf]

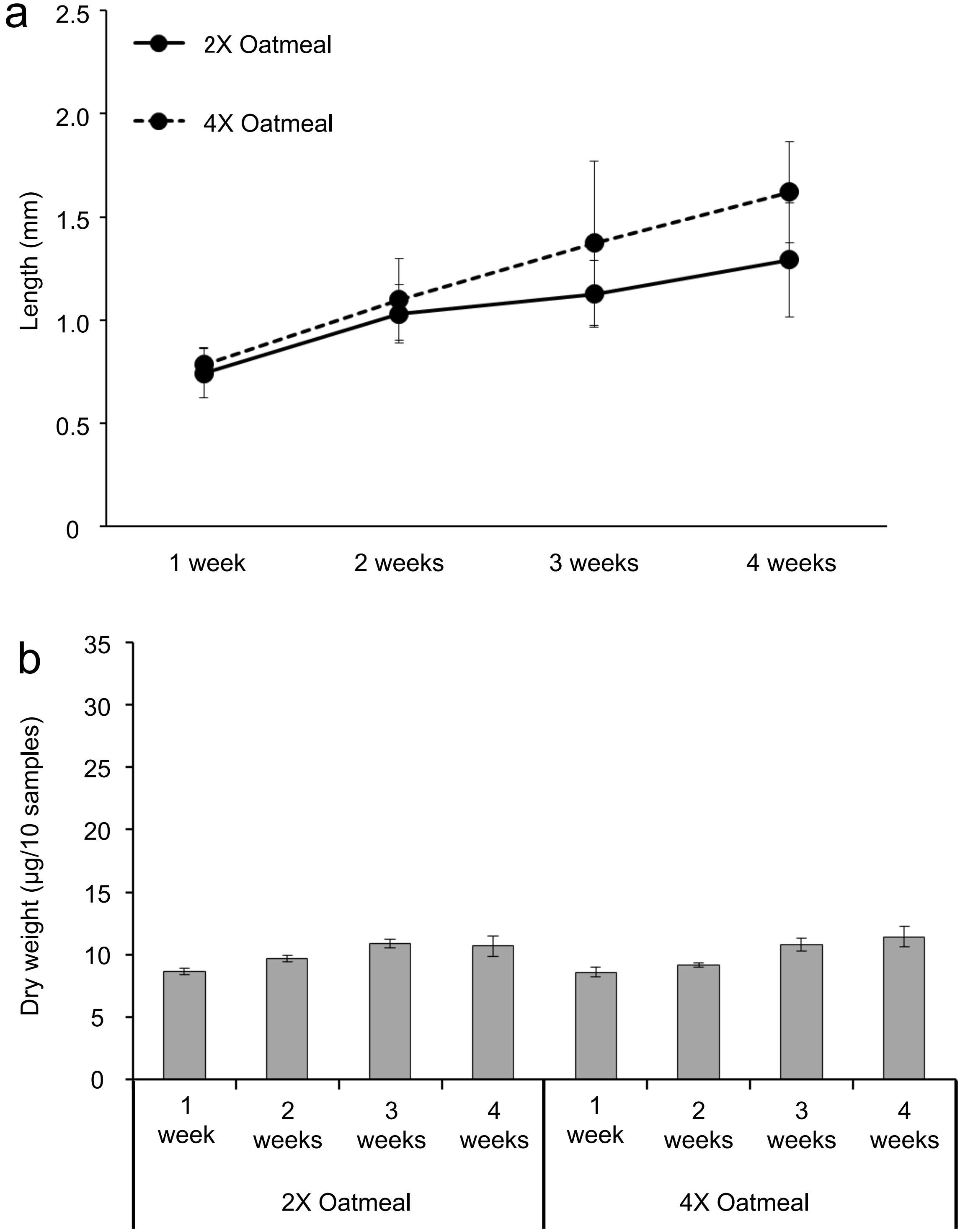

Additional file 4. Length and dry weight of symbiotic protocorm under the conditions with high concentration of oatmeal.

(a) The length of symbiotic protocorms under the 2X- and 4X-strength oatmeal for four weeks after seeding. (b) The dry weight of symbiotic protocorm under the 2X- and 4X-strength oatmeal conditions for four weeks after seeding. Error bars of (a) and (b) represent the standard errors of the mean in five and three biological replicates, respectively.
