# Supplementary material for: Quantitative evaluation of protocorm growth and fungal colonization in Bletilla striata (Orchidaceae) reveals less-productive symbiosis with a non-native symbiotic fungus
Source: BMC Plant Biol. 2017 Feb 21;17:50. doi: 10.1186/s12870-017-1002-x (PMC5320772; doi:10.1186/s12870-017-1002-x)
Supplement: Additional file 5: — Growth of fungal hyphae on oatmeal agar medium. The hyphae of the symbiotic fungi on oatmeal agar media with (a) 1×-, (b) 2×-, and (c) 4×- strength oatmeal stained by 0.05% trypan blue solution. Scale bars, 500 μm. The experiment was repeated five times with similar results. (PDF 977 kb) [file 12870_2017_1002_MOESM5_ESM.pdf]

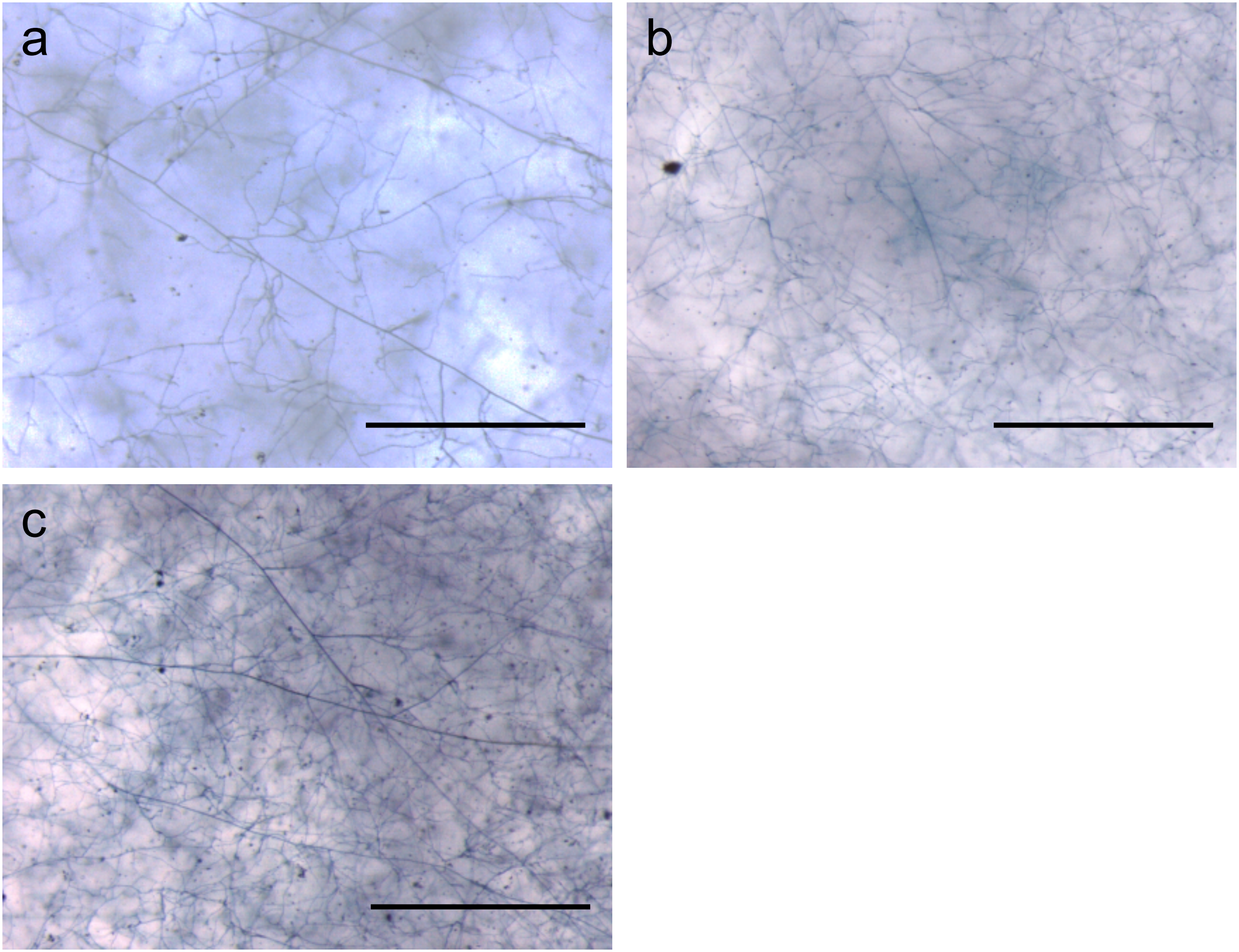

Additional file 5. Growth of fungal hyphae on oatmeal agar medium.
The hyphae of the symbiotic fungi on oatmeal agar media with (a) 1X-, (b) 2X-, and (c) 4X-strength oatmeal stained by 0.05 % trypan blue solution. Scale bars, 500 μm. The experiment was repeated five times with similar results.
