# Supplementary material for: Quantitative evaluation of protocorm growth and fungal colonization in Bletilla striata (Orchidaceae) reveals less-productive symbiosis with a non-native symbiotic fungus
Source: BMC Plant Biol. 2017 Feb 21;17:50. doi: 10.1186/s12870-017-1002-x (PMC5320772; doi:10.1186/s12870-017-1002-x)
Supplement: Additional file 6: — Quantitative evaluation of symbiotic cells in Pecteilis radiata protocorm. (a) Symbiotic cells with hyphal coils in P. radiata protocorm. Scale bars, 50 μm. (b) Ratio of the number of symbiotic cells at each stage in a symbiotic protocorm. Each value represents the average number of symbiotic cells in ten protocorms. The experiments were repeated six times with similar results. (PDF 959 kb) [file 12870_2017_1002_MOESM6_ESM.pdf]

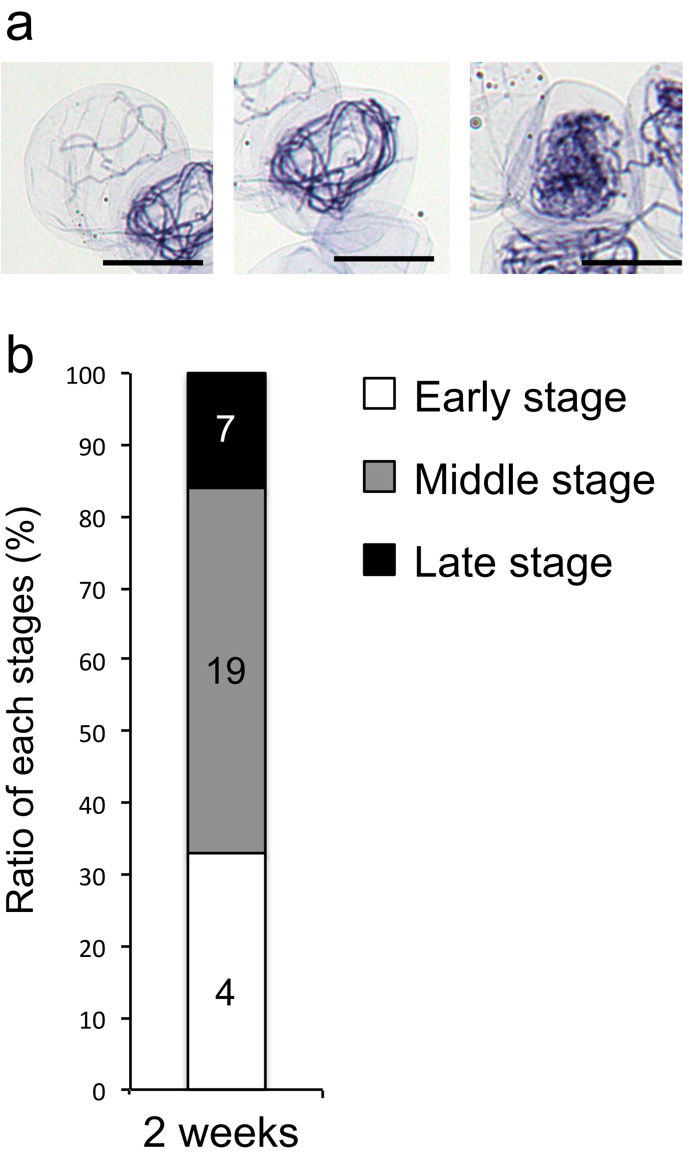

Additional file 6. Quantitative evaluation of symbiotic cells in *Pecteilis radiata* protocorm.

(a) Symbiotic cells with hyphal coils in *P. radiata* protocorm. Scale bars, 50 μm. (b) Ratio of the number of symbiotic cells at each stage in a symbiotic protocorm. Each value represents the average number of symbiotic cells in ten protocorms. The experiments were repeated six times with similar results.
