# Supplementary material for: Quantitative evaluation of protocorm growth and fungal colonization in Bletilla striata (Orchidaceae) reveals less-productive symbiosis with a non-native symbiotic fungus
Source: BMC Plant Biol. 2017 Feb 21;17:50. doi: 10.1186/s12870-017-1002-x (PMC5320772; doi:10.1186/s12870-017-1002-x)
Supplement: Additional file 7: — Quantitative evaluation of symbiotic cells in a protocorm under conditions with high concentrations of oatmeal. (a) The number of symbiotic cells per one protocorm under 2×- and 4×-strength oatmeal conditions for four weeks after seeding. (b) Ratio of the number of symbiotic cells at each stage in a symbiotic protocorm under 2×- and 4×-strength oatmeal conditions. Error bars represents the standard error of the mean in ten protocorms. The experiments were repeated three times with similar results. (PDF 140 kb) [file 12870_2017_1002_MOESM7_ESM.pdf]

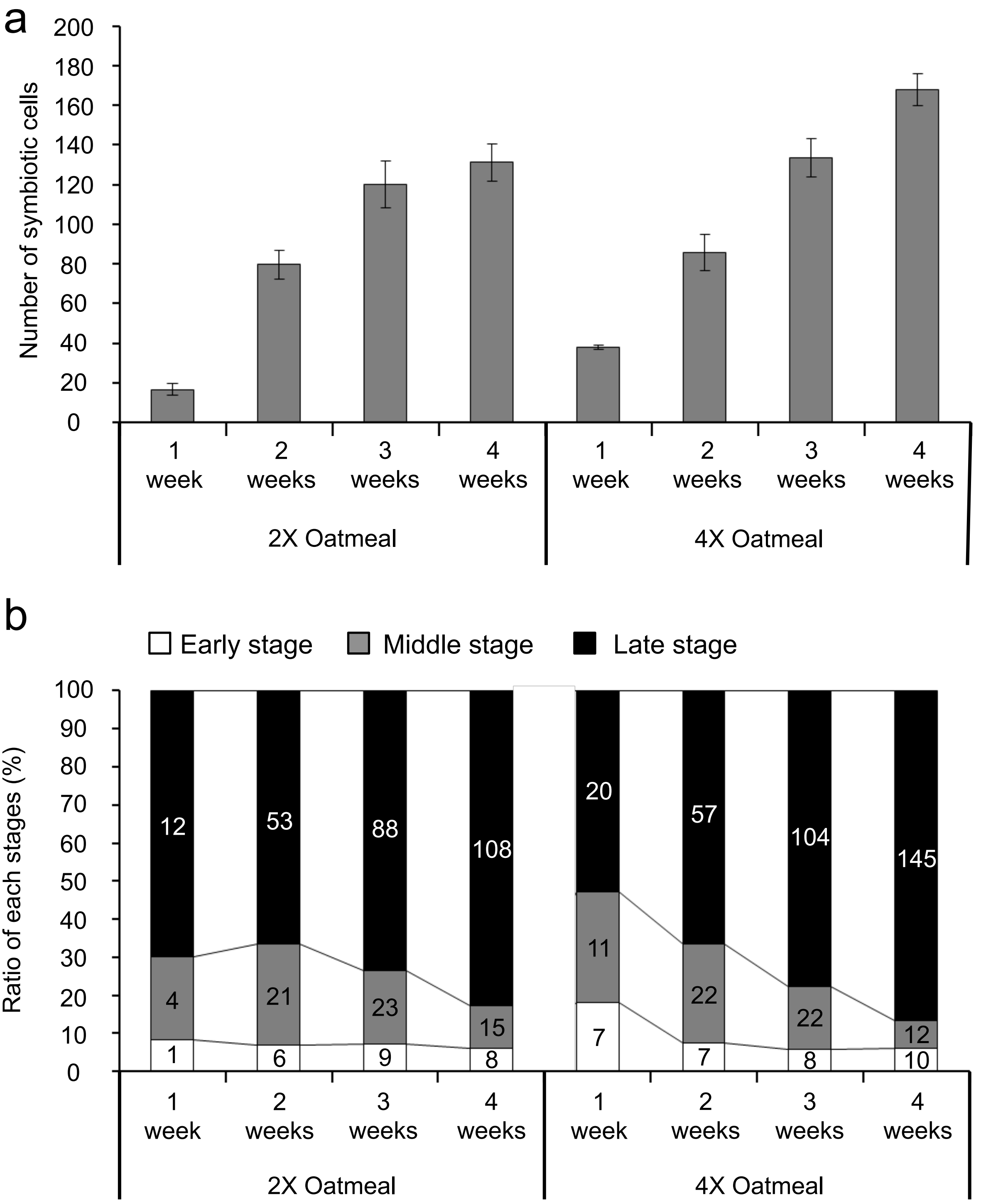

Additional file 7. Quantitative evaluation of symbiotic cells in a protocorm under conditions with high concentrations of oatmeal.

(a) The number of symbiotic cells per one protocorm under 2X- and 4X-strength oatmeal conditions for four weeks after seeding. (b) Ratio of the number of symbiotic cells at each stage in a symbiotic protocorm under 2X- and 4X-strength oatmeal conditions.

Error bars represents the standard error of the mean in ten protocorms. The experiments were repeated three times with similar results.
